# Clonal Cocoa Varieties Growth and Leaf Non‐Structural Carbohydrate Response to Field Stress Conditions

**DOI:** 10.1002/pei3.70160

**Published:** 2026-05-13

**Authors:** Moses Kwame Aidoo, Esther Anokye, Priscilla Araba Amissah, Atta Ofori, Francis Kwame Padi

**Affiliations:** ^1^ Physiology/Biochemistry Division Cocoa Research Institute of Ghana New Tafo‐Akim Eastern Region Ghana; ^2^ Plant Breeding Division Cocoa Research Institute of Ghana New Tafo‐Akim Eastern Region Ghana

**Keywords:** environmental stress, metabolism, non‐structural carbohydrate, pod numbers

## Abstract

This study investigated the growth and leaf metabolism of clonal cocoa varieties in response to field stress conditions during wet and dry seasons. It was hypothesized that clonal cocoa varieties differ in their growth, photochemical efficiency, relative water content and central carbon metabolism under seasonal field stress conditions. Fourteen cocoa clone varieties between 35 and 44 months after planting were evaluated. Using various standard procedures in the field and laboratory, the plants were assessed in both wet (as unstress condition) and dry (as stress condition) seasons field conditions. Soil moisture content, photochemical efficiency, relative water content, non‐structural carbohydrate, carbon, nitrogen, growth and pod numbers were measured and compared among the seasons. The clones conserved cellular water, and this manifested in high levels of relative water content. Photochemical efficiency reduced in most of the clonal cocoa varieties during the dry season. Soluble sugars, starch and non‐structural carbohydrate accumulated in the leaves of the plants during the dry season. The levels of carbon and nitrogen were significantly high and low respectively in the dry season. Some clonal cocoa varieties increased growth rate and had a greater number of pods. The correlation analysis revealed a relationship among non‐structural carbohydrate, growth and physiological traits. Clone varieties CRG 0132/105, CRG 0145/205, CRG 0314/102 and T65/238 were the most tolerant in response to field stress conditions, and this reflected in the accumulation of soluble sugars, starch and non‐structural carbohydrate, improved photosynthetic efficiency, conserved cellular water and enhanced growth rates and pod numbers agreeing to the hypothesis.

## 1 Introduction

Cocoa seedlings derived from seeds are mainly used for cultivation of cocoa plantations in West Africa. But adoption of clones planting materials in cocoa production is recently gaining importance in the subregion (Ofori et al. 2016). However, there is dearth of information on the adaptation of clonal cocoa materials to the extreme environmental conditions such as poor soil fertility, high temperatures, heat and drought stress in the subregion (Ofori et al. 2024). These stresses have contributed to the difficulties in establishing and sustaining cocoa plantations to ensure high productivity (Padi et al. 2015). Field stress conditions cause several events in the plants including loss of leaf water content and turgor losses that affect plant height and stem diameter growth, ion uptake and plant metabolism (Oguz et al. 2022). Drought and high temperature induced osmotic stress which activate physiological changes in clonal cocoa plants. A common response to this activation is stomatal closure which reduces water losses, leading to sharp reduction of photosynthesis and other gas exchanges indicating reduced CO_2_ assimilation (Zambrano et al. 2021). Under stress conditions, photochemical efficiency reduced, and clones that maintained higher leaf relative content and recovery from photochemical efficiency after rewatering are classified as plants that exhibited physiological resilience (Zambrano et al. 2021). Plants have evolved various stress tolerant strategies to survive in the field. These strategies include changes in the levels of morphology, physiology and alteration of non‐structural carbohydrate (Larkunthod et al. 2018; Aidoo et al. 2024). However, knowledge on the effects of changing field stress conditions such as water deficit and high temperature on the growth and metabolism of clonal cocoa varieties are scarce (Lahive et al. 2019).

Plant soluble sugars and starch are the constituents of non‐structural carbohydrate (NSC) which are the main photosynthates and play a crucial role in regulating plants' physiological modifications in response to challenging field conditions (Ai et al. 2017). Non‐structural carbohydrates are among the most accumulated solutes in plant tissue under drought and extreme temperature conditions (Guo et al. 2022). The accumulation of a particular or individual solute contributes slightly to stabilizing enzymes, membranes and other cellular components in many species (Nawaz et al. 2025). These cellular protections are mainly enormous when there is an accumulation of a multitude of solutes (Silva et al. 2010). Plant cellular protection during environmental stress requires metabolism or uptake of solutes. This process is generally slow and sensitive to the timing and intensity of environmental stress (Sanders and Arndt 2012). The quality and allocation of non‐structural carbohydrates shift when plants are exposed to field stress conditions. These exposures reduce photosynthesis and growth and hasten the accumulation of non‐structural carbohydrates within plants (He et al. 2023). Field stress conditions negatively influence the stability of carbon uptake, consumption and deprive plants of soluble sugars and starch requirements for growth and defense strategies (Tomasella et al. 2019). Starch accumulation in response to field stress has been reported to protect the cellular organs by maintaining membrane hydration, turgor and regulating stomatal closure to conserve water (Varshney et al. 2023). Starch is also known to hydrolyze and remobilize after plants' exposure to field stress conditions to supply sugars for photosynthetic functions (Zhang et al. 2020). Sugars, on the other hand, accumulate in plants during stress to regulate cells' osmotic potential to maintain cell turgidity (Vuerich et al. 2023). Plants that survive and maintain high levels of stem water potential under field stress conditions accumulate high levels of non‐structural carbohydrates (O'Brien et al. 2014). Therefore, there is the need to understand and select clonal cocoa varieties that are efficient in accumulating sugars and starch, which can be enhanced to improve the resilience of tree crops in response to field stress conditions.

Metabolism of carbon and nitrogen is a basic plant process that is involved in the growth, development and survival of plants under environmental stress conditions (Rachmilevitch et al. 2008). The levels of non‐structural plant carbohydrate are indicative of the magnitude of carbon and nitrogen levels in the plant tissues (Liu et al. 2018). This regulates the metabolism and growth of plants in response to changing field stress conditions (Coruzzi and Zhou 2001). Regular allocation of carbon and nitrogen improves plant tolerance to environmental stresses (Huang and Fu 2000). Field stress conditions deplete stored carbohydrate and induce carbon starvation, resulting in mortality of plants (McDowell et al. 2022). Carbon starvation can worsen cellular water movement; therefore, sufficient NSC levels in the secondary phloem can prevent phloem turgor losses and leakage of xylem water (Sevanto et al. 2014). Availability of carbon enhances metabolite production, which is essential for plant defenses and tolerance when exposed to abiotic stress in the field (Nardini et al. 2013). Nitrogen is an important factor in determining the growth, survival and productivity of plants. The element is also associated with plant adaptations to field stress conditions (Chen et al. 2012). For instance, plants that are susceptible to field stress conditions require the application of more fertilizer to be tolerant (Zhu 2016). Nitrogen enhances root growth and development, and this increases root number and surface area, improving plant water uptake (Li et al. 2009). These are mechanisms that are associated with stomata opening, enhancing CO_2_ fixation and assimilation in the plant leaves during field stress conditions (Ren et al. 2015). Plants mitigate drought effects using metabolic activities even at low tissue water potential (Aoyama et al. 2014). Regulation of plant growth and development is associated with the balance between carbon and nitrogen metabolism ratio in plant cells (Coruzzi and Zhou 2001). The carbon/nitrogen balance is very important in controlling leaf senescence, which can be activated by high carbon and low nitrogen activity (Aoyama et al. 2014). However, metabolism and allocation of carbon and nitrogen in clonal cocoa plant leaves exposed to extreme field conditions have hardly been explored.

To understand these processes, vegetatively generated (from high vigor and yield‐combining abilities parent) clonal cocoa varieties were selected and investigated in this study. The main parentage or source of the clones is Ghana, Trinidad, and Parinari origin, belonging to the Marañón genetic group (Padi et al. 2015). In evaluating 116 cocoa clones introduced into Ghana over different periods in the field conditions, Ofori et al. (2016) revealed genetic variation among agronomic traits and yield performance. The group then concluded that the combination of yield with agronomic traits such as stem diameter increment, number of beans per pod, beans weight, and pod value reflected the potential cocoa yield improvement. Cocoa clones' combining ability, bean weight, and their genetic factors that influence them have been shown as important predictors of yield under field conditions (Ofori et al. 2024). Cocoa clones from diverse backgrounds have been found to exhibit lower disease incidence and better yield retention under field conditions (Ofori et al. 2022). The change of soil water and precipitation, which resulted in drought stress, has been reported to have reduced cocoa yield (Asitoakor et al. 2022). However, few cocoa clonal varieties have been investigated in the field to understand their adaptation (Benjamin et al. 2025). Therefore, it is essential to elucidate and understand the strategies that involve plant height, stem diameter, and leaf metabolism of clonal cocoa varieties in response to extreme field conditions to complement future cocoa breeding programmes for adaptation.

The goal of this study is to investigate the growth and leaf metabolism of clonal cocoa varieties in response to high temperature, low rainfall and soil moisture deficit (field stress conditions) during the dry season and compared to optimum temperature, high rainfall and soil moisture content (field unstress condition) mainly experienced in the wet season. It was hypothesized that clonal cocoa varieties differ in their growth, photochemical efficiency, relative water content and central carbon metabolism under seasonal field stress conditions. The study highlights additional knowledge towards unraveling non‐structural carbohydrate, carbon and nitrogen metabolism and growth of clonal cocoa plants and how these affect pod numbers in response to extreme field conditions to improve cocoa breeding programme.

## 2 Materials and Methods

### 2.1 Experimental Design, Climatic Data and Planting Materials

**FIGURE 1:**
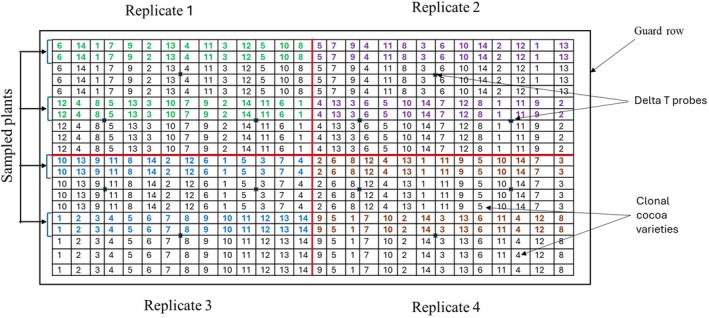
The schematic diagram showing strip design used for the study. Colored numbers represent the clonal cocoa varieties that were measured and sampled for the physiological traits and non‐structural carbohydrates respectively. All plants were measured for the determination of growth (plant height and stem diameter). Red line indicating the separation of the replications. In each of the replicates were three delta probes installed for measuring soil water content.

**FIGURE 2:**
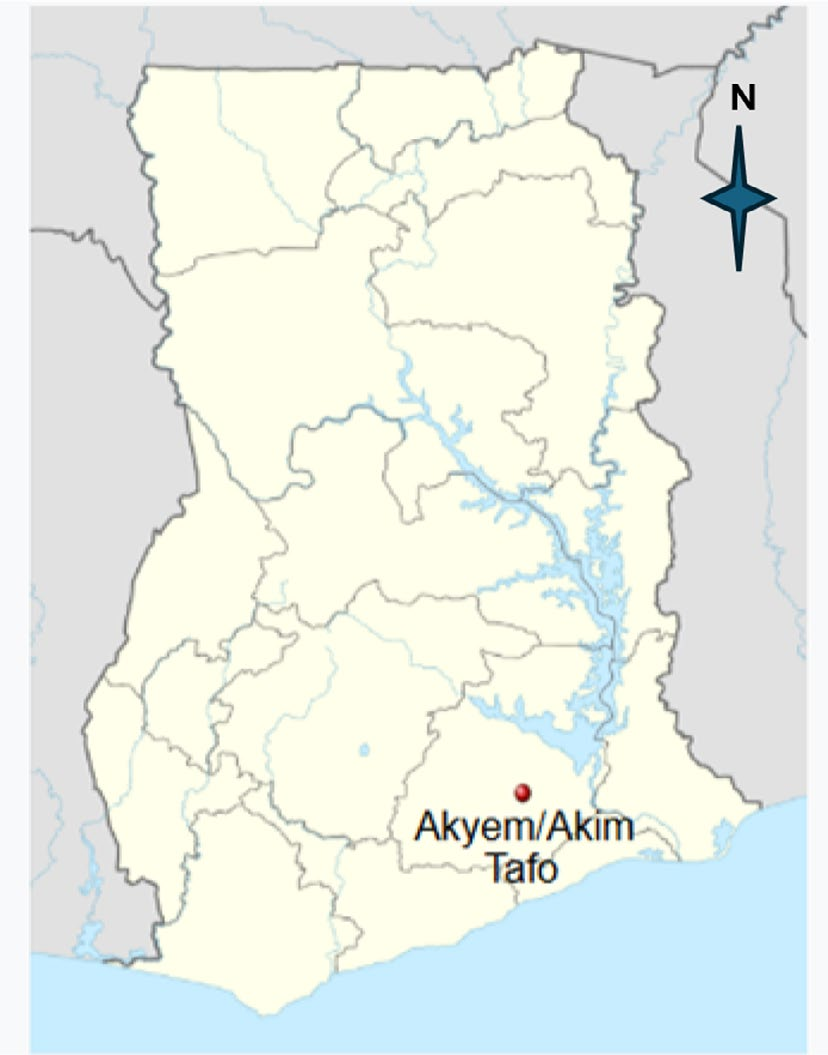
A Ghana map showing the location (New Tafo‐Akim) of the study with geographic coordinates (Wikipedia 2026).

The experiment was laid out using randomized complete block design (RCBD) in strip planting procedure and replicated 4 times (Figure 1) at the research plot of Cocoa Research Institute of Ghana (CRIG) in June 2019. The location of the experiment was New Tafo‐Akim with a geographic coordinate of 6°13′19″N 0°21′56″W, Eastern Region of Ghana with an elevation of approximately 233 m above sea level. The location experienced a tropical savannah climate with high temperatures reaching from 32°C to 36°C (Wikipedia. 2026). The annual rainfall recorded within 6 years ranged from 800.10 to 1782.10 mm (Figure SD3b). The soil type was sandy loam (Table SD1). Planting distance was 3 m x 3 m and each plot consisted of 14 of each of the clonal cocoa varieties (Figures 1 and 2). Data collection was carried out in 2022 and 2023 growing cycle during wet (from May to October thus 35–39 months after planting) and dry (from November to April, thus 40–44 months after planting) seasons (Figure 2).

The origin of the clones used in this study is Parinari and Iquitos belonging to Marañón genetic group and derived from PA 35 × NA 32, PA 7 × IMC 47 and PA 7 × NA 32 made in Trinidad and collected by Posnette in 1944 and GU 144C × EQX 3338 made in Ghana (Padi et al. 2015). The clonal materials were generated vegetatively (budding). Apart from T65/238, all clones prefixed CRG were developed through ortet selection under various CRIG breeding programmes. T65/238 was developed from a selection of seedlings from a cross between clone PA 7 (a clone of the Marañón genetic group) and IMC 53 (Iquitos and Marañón clone genetic group). The clones were selected for this study based on the following characteristics of their sources; ease of establishment, vigor, precocity and high dry bean yields (Padi et al. 2015) to ascertain their physiology, growth and metabolism responses under field stress conditions.

### 2.2 Data Collection

Soft selection traits (plant height, stem diameter, and pod number) and hard selection traits (soil moisture content, ambient temperature, rainfall, Fv/fm, relative water content, soluble sugar, starch, non‐structural carbohydrate, soluble sugar starch ratio, carbon, nitrogen, and carbon nitrogen ratio) were investigated to answer and test the research questions and hypothesis respectively. Soft selection traits involve parameters that evaluate how plants compete for resources like water, sunlight, and nutrients in the environment while hard selection traits reflect the plant physiological capacity and energy to survive environmental stress (Bell et al. 2021).

### 2.3 Soil Moisture Content, Rainfall, and Ambient Temperature

Three PR2/6 delta T probes with a sense element at 10, 20, 30, 40, 60 and 100 cm (Delta‐T Devices Ltd., Cambridge, CB25 0EJ, United Kingdom) were installed at 1.5 m from cocoa plants in each of the replicates to assess soil moisture content during the experimental period. PR2 profile probe has been found to be a reliable alternative to more expensive and difficult techniques and at a level that has been accepted as accurate for research (Dhakal et al. 2019). The amont of rainfall and ambient temperature data that were used as comparison of the study were obtained from CRIG meteorological Station at New Tafo‐Akim, Eastern Region of Ghana.

### 2.4 Plant Height and Stem Diameter Growth Rate

Plant height and stem diameter growth rates were estimated from the initial measurement and the final measurement after 6 months interval using meter rule and electronic digital vernier calipers (Factory IP67, China) for plant height and stem diameter respectively. Plant height was measured from the ground level to the canopy top and stem diameter from 15 cm above the bud union. The period of which plant height and stem diameter were taken spanned within the study period. Plant height and stem diameter growth rates were estimated using final height or diameter, initial height or diameter, final and initial time. The plant height was measured in cm, stem diameter in mm per day and converted to percentage as expressed in the following formulas:

(1) $$P\mathrm{HGR}=\frac{H2-H1}{T2-T1}\times 100$$

where PHGR is plant height growth rate, H2 is final height, H1 is initial height, T2 is final time and T1 is initial time all expressed in percentage.

(2) $$\text{SDGR}=\frac{D2-D1}{T2-T1}\times 100$$

where SDGR is stem diameter growth rate, D2 is final diameter, D1 is initial diameter, T2 is final time, and T1 is initial time, all expressed in percentages.

### 2.5 Physiological and Yield Studies

The maximum quantum efficiency of photosystem II (PSII) photochemistry (Fv/Fm) was determined using a fluorescence meter (Fluorpen FP 110, Drásov, Czech Republic). Prior to measurements, four fully expanded leaves from two plants per variety per replicate were dark‐adapted for 30 min using leaf clips to ensure complete oxidation of PSII reaction centres.

Relative water content was determined as described by Pieczynski et al. (2013). Ten leaf discs cut from fully expanded leaves harvested from the cocoa clone plants were immediately weighed to determine fresh mass (FM). The leaf samples were then floated on distilled water in a 50 mL falcon tube and incubated in a refrigerator for 24 h to allow full rehydration. After hydration, the leaf discs were gently blotted with tissue paper to remove excess moisture on the surface and weighed to obtain turgid mass (TM). Subsequently, the samples were oven dried at a temperature of 65°C for 3 days until a constant mass was achieved to determine the dry mass (DM). Relative water content (RWC) was then calculated using the formula below and parameters (fresh mass, turgor mass and dry mass of 4 sample leaves harvested from two trees per variety per replicate).

(3) $$\mathrm{RWC}=\frac{\mathrm{FM}-\mathrm{DM}}{\mathrm{TM}-\mathrm{DM}}\times 100$$

where RWC is relative water content, FM is fresh mass, TM is turgor mass, and DM is dry mass of sampled leaf. Number of pods observed during the period of the study were counted at wet and dry seasons.

### 2.6 Sampling and Carbohydrates Analyses

Sampling procedure and non‐structural carbohydrate were determined as described in detail by Landhäusser et al. (2018). The enzymatic activity of the sampled leaves (newly fully expanded leaves—third after the flashes) was immediately stopped. This was done by cutting the leaves into smaller pieces and boiling 0.5 g in 80% ethanol under reflux for 30 min. The supernatant separated into a new flask after boiling. The ethanol was then evaporated under reduced pressure using a rotary evaporator. Two milliliters of water were added to dissolve all sugars in the solution. The solution was then filtered into clean flasks using Whatman No. 41 filter paper. The filtered solution was clarified by removal of non‐sugar components using a solution of 0.3 N Ba(OH)_2_ and 5% ZnSO_4_ solution. The resulting solution was shaken with cation and anion exchange resins to remove inorganic ions and acids present in the solution through Whatman No. 41 filter paper. The volume of the filtrate was poured into vials and labeled as soluble extract (SE) for further analysis. The residue that remained after the removal of 80% ethanol was digested under reflux for 1 h using 25 mL of 1.5 N sulfuric acid. The solution was filtered and the residue washed with water several times. The acid in the solution was neutralized by adding solid BaCO_3_. The solution was centrifuged at high speed (26,000×g) to remove precipitate, filtered, and shaken with ion exchange resins to deionize the solution. The filtered solution was poured into vials and labeled as acid extract (AE). In the analysis, 1 mL of solutions (SE and AE) was added to 1 mL 10% phenol reagent in a test tube, mixed. To the solution in the test tube, 5 mL of concentrated sulfuric acid was added straight and mixed thoroughly and leave to cool. The sugar and starch were analyzed by reading absorbance on the spectrophotometer (American Laboratory Trading, USA) at 490 nm. Non‐structural carbohydrates were then estimated from soluble sugars and starch.

### 2.7 Determination of Leaf Nitrogen and Carbon Contents

Oven dried leaf samples at the temperature of 65°C for 3 days were ground using TissueLyzer (RetschGmbh and Co.). The ground samples were analyzed for nitrogen and carbon contents using Kjeldahl (Bremner 1965) and Walkley and Black (1934) wet oxidation methods, respectively. In determining nitrogen, approximately 0.5 g of the ground leaf sample was weighed into a Kjeldahl digestion tube, followed by the addition of 10 mL concentrated sulfuric acid (H_2_SO_4_) and catalyst mixture (commonly potassium sulfate and copper sulfate) to accelerate digestion. The samples were digested at 350°C–420°C in a digestion block until the solution became clear. After cooling, the digest was diluted with distilled water and transferred to a distillation unit. The digested solution was made alkaline by adding 40% sodium hydroxide (NaOH), which converted ammonium ions into ammonia (NH_3_). The released ammonia was distilled and captured in a receiving flask containing boric acid solution with a mixed indicator. The trapped ammonia was then titrated with standard hydrochloric acid (HCl) or sulfuric acid (H_2_SO_4_) to determine the amount of nitrogen present in the sample.

For leaf carbon determination, 0.5 g of finely ground sample was accurately weighed and transferred into a conical flask. To the sample, 10 mL of 1 N potassium dichromate (K_2_Cr_2_O_7_) solution was added, followed by 20 mL of concentrated sulfuric acid (H_2_SO_4_). The mixture was gently swirled to ensure thorough mixing and allowed to stand for 30 min to facilitate oxidation of organic carbon by dichromate under acidic conditions. After the oxidation reaction, 200 mL of distilled water was added to dilute the mixture. Subsequently, 10 mL of phosphoric acid (H_3_PO_4_) and 1 mL of diphenylamine indicator were added. The excess potassium dichromate was then titrated with 0.5 N ferrous ammonium sulfate (Fe(NH_4_)_2_(SO_4_)_2_) until the color changed from violet‐blue to green, indicating the end point. A reagent blank sample was processed simultaneously with plant materials. The percentage of carbon in the sample was calculated using the following equation.

(4) $$\text{Carbon}=\frac{\mathrm{Vb}-\mathrm{Vs}\times N\times 0.003\times 100}{M}\times 1.3$$

where Vb is the volume of ferrous ammonium sulfate used for the blank titration (mL). Vs is the volume of ferrous ammonium sulfate used for the sample titration (mL). N is the normality of ferrous ammonium sulfate solution. M is the mass of the sample. 0.003 is the equivalent weight of carbon (g) oxidized by 1NK_2_Cr_2_ O_7_ and 1.33 is the correction factor to account for incomplete oxidation of organic carbon.

### 2.8 Determination of Soil Properties

Physical and chemical soil analysis were conducted on soils sampled from 0 to 20 cm depth on each of the plots before planting (Table SD1). Soil pH was measured potentiometrically in a slurry using an electronic pH meter (SevenCompact Duo pH/conductivity, Mettler Toledo, US). Ammonium acetate methods (Black 1965) and Mehlich (1984) were used to extract exchangeable bases and available P, respectively. The detail of the extraction of organic carbon (Walkley and Black 1934) and total nitrogen (Bremner 1965) can be found under leaf extraction at materials and method of this study.

For the determination of exchangeable bases, air‐dried soil samples were gently crushed and passed through a 2 mm sieve to remove debris and stones. Approximately 5 g of soil was weighed into a 100 mL extraction flask. Subsequently, 50 mL of 1 M ammonium acetate solution (pH 7.0) was added to the soil sample. The mixture was shaken on a mechanical shaker for 30 min to allow ammonium ions to replace exchangeable base cations on the soil colloids. After shaking, the suspension was filtered through Whatman No. 42 filter paper into a clean container. The filtrate containing the displaced cations was used for the determination of exchangeable bases. Potassium (K) concentration was determined using a flame photometer, while calcium (Ca) and magnesium (Mg) were measured using an atomic absorption spectrophotometer (AAS, Chongqing Drawell Instrument Co. Ltd., Chongqing, China).

For the determination of phosphorous (P) approximately 2 g of air‐dried soil samples ground and passed through 2 mm sieve was weighed into a 50 mL extraction bottle. Then 20 mL of Bray 1 extracting solution (0.03 M NH4F + 0.025 M HCl) was added to the soil sample. The suspension was shaken for 1 min on a mechanical shaker and immediately filtered through Whatman No. 45 filter paper. An aliquot of the filtrate was then used for phosphorus determination by the molybdenum blue colorimetric method. The color was developed by adding ammonium molybdate and stannous chloride reagents forming a blue complex. The absorbance of the solution was measured at approximately 660–882 mm using a spectrophotometer. Available P concentration was calculated using a standard calibration curve prepared from known phosphorus standards.

### 2.9 Data Analysis

ANOVA was performed to explore the effects of growth, physiology, seasonal carbohydrate, and carbon‐nitrogen metabolism of the varieties using GENSTAT 9th Edition. The means were separated by Fisher pairwise comparison and student *t*‐test at *p* ≤ 0.05 to identify differences between the means of the individual varieties and the sampling time points (growing dry and wet seasons).

## 3 Results

### 3.1 Hard Selection Traits

#### 3.1.1 Soil Moisture Content, Properties, Ambient Temperature and Rainfall

To understand the soil moisture levels in which the varieties were grown, soil moisture content was determined. The soil moisture assessed at 10 to 40 cm depth in the plots followed a similar trend. The soil 10 cm depth moisture ranged from 8.86% in December 2022 to 22.95% in June 2022. At 40 cm depth the values recorded ranged from 17.31% to 28.28% in December 2022 and June 2022 respectively (Figure SD1). These levels of soil moisture across soil depths were higher during the wet season compared to the dry season of an average of 23.67% and 18.23% respectively (Table SD3). The soil texture of the experimental site was sandy loam with a varied amount of sand (1.5%), clay (0.5%) and silt (1.0%, Table SD1). Sand content across the soil depths was high with an average of 77.24% compared to clay and silt which had 13.76% and 9% respectively (Table SD1). Soil carbon content, available P and exchangeable K are the soil chemicals that fell below the critical levels for cocoa cultivation (Table SD1). However, across soil depth maximum levels recorded for pH, total nitrogen, exchangeable magnesium and exchangeable calcium were high then the critical values (Table SD2). The soil moisture monitored before and during the experiment showed variations in the 10 and 40 cm soil depths and the months (Figure SD1). Temperatures were mainly low and high during wet and dry seasons respectively. In June 2022 which serves as a time point for taking data at the wet season had higher rainfall compared to the other months. December 2022 as the dry season time point had lower rainfall (Figure SD2). The temperature and rainfall recorded during September 2022 intercepted with each other (Figure SD2). Several variations were observed in monitoring of rainfall and temperature before, during and after the experiment (Figure SD3a,b).

#### 3.1.2 Fv/Fm and Relative Water Content of Clonal Cocoa Varieties

**FIGURE 3:**
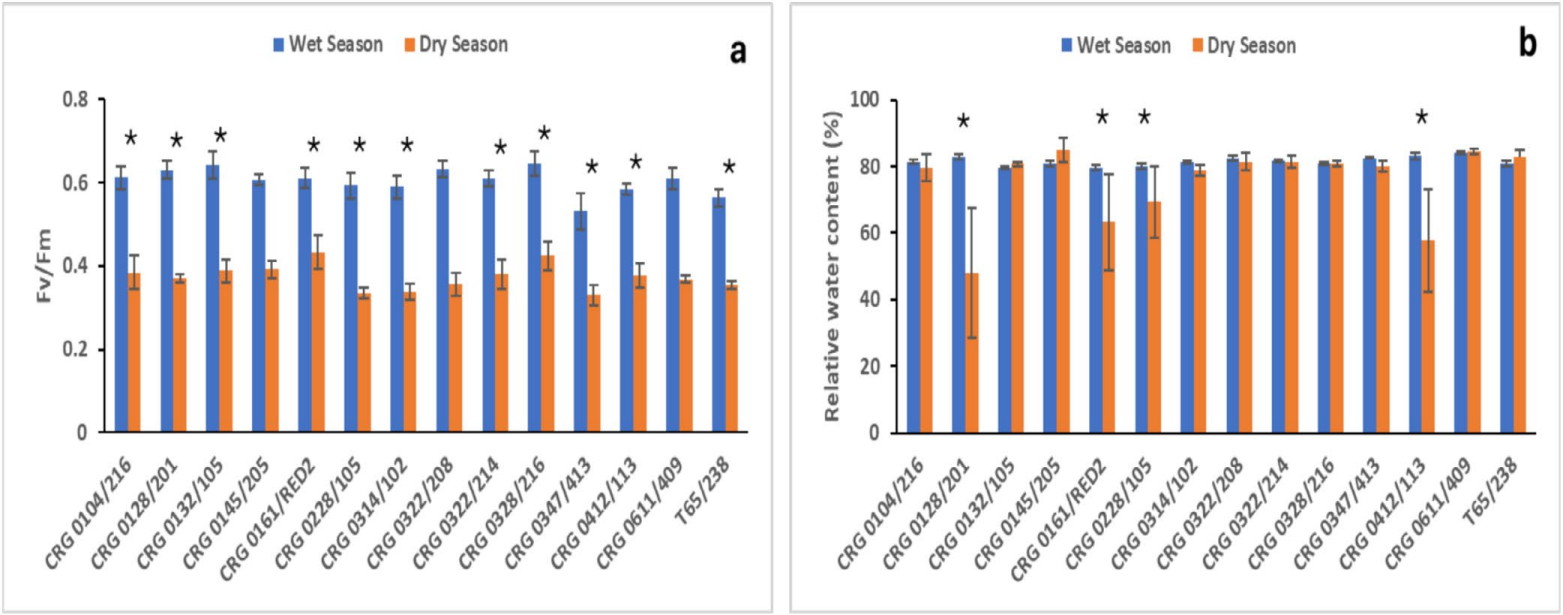
Photochemical efficiency measured as Fv/Fm (a) and relative water content (b) of some clonal cocoa varieties in response to field stress conditions during the vegetative phase recorded in 2022 and 2023 wet and dry growing seasons between 35 and 44 months after planting. Values are the means of 4 replicates with standard error bars. Bars with asterisks indicate significance (*p* = 0.005, student *t*‐test) between wet and dry seasons.

The Fv/Fm measured to understand the photosystem II efficiency was generally high (*p* ≤ 0.001) during the wet season relative to the dry season when soil moisture content was low (Figure 3a, Table 1). Showing a varietal significant effect (*p* = 0.014), Fv/Fm ranged from 0.56 to 0.65 in CRG 0347/413 and CRG 0328/216 respectively during the wet season, and in the dry season ranged from 0.33 in both clones CRG 0347/413 and CRG 0228/105 to 0.43 in CRG 0161/RED2 (Figure 3a, Table 1). The relative water content detected in clones was significantly different (*p* = 0.047) regarding the interaction between season and variety (Table 1, Figure 3a). The values recorded were mostly lower during the dry season relative to the wet season (*p* = 0.007) when the soil moisture contents were high (Figure 3b). Clones CRG 0128/201, CRG 0228/105, CRG 0161/RED2, and CRG 0412/113 decreased their relative water contents with reduced soil moisture content during the dry season (Figure 3b). The maximum values of Fv/Fm across the varieties were high during the wet season compared to the dry season while relative water content was higher during the dry season compared to the wet season. However, in considering the median, minimum, and mean levels of relative water content, high values were observed during the wet season compared to the dry season (Tables SD4 and SD5).

**TABLE 1:** Summary of source of variation from clone cocoa varieties, seasons and interaction for all parameters.

Factor	Fv/Fm	Chl C	RWC	SS	S	NS	SS/S	C	N	C/N	NP
Variety	2.24*	1.61^a^	1.74^a^	1.60^a^	2.68**	2.68**	1.21^a^	0.86^a^	1.34^a^	0.96^a^	4.29**
Season	543.03**	2.62^a^	7.78**	45.65**	30.57**	1.66^a^	69.92**	7.70**	4.78*	11.17**	7.05**
V × S	0.54^a^	0.68^a^	1.86*	3.45**	4.33**	4.88**	1.99*	0.25^a^	0.57^a^	0.42^a^	0.18^a^

*Note:* Values are *F*‐values. Values with asterisks are significantly different (*p* ≤ 0.05).

Abbreviations: C, carbon; C/N, carbon nitrogen ratio; Chl C, relative chlorophyll content; Fv/Fm, photochemical efficiency; N, nitrogen; NP, number of pods; NS, non‐structural carbohydrate; RWC, relative water content; S, starch; SS, soluble sugars; SS/S, soluble sugars starch ratio; V × S, variety × season interaction.

* Significance at 0.05.

** Significance at 0.01.

^a^ Non‐significant.

#### 3.1.3 Leaf Carbohydrate Metabolism of Clonal Cocoa Varieties

The change of soluble sugars was significantly high (*p* ≤ 0.001) in the dry season compared to the wet season except in clone CRG 0412/113 which had the least 21.39 mg g^−1^. The trait also showed variety × season interaction (Tables 1 and 2). The top three clones which accumulated high soluble sugars during the dry season included CRG 0161/RED2 (53.11 mg g^−1^), T65/238 (52.39 mg g^−1^), and CRG 0228/105 (40.79 mg g^−1^, Table 2). The starch content which showed variety × season interaction was generally low during the dry season (*p* ≤ 0.001) compared to the wet season and significantly ranged from 12.27 to 33.62 mg g^−1^ in CRG 0412/113 and CRG 0161/RED2 respectively (Tables 1 and 2). However, comparing the clones during the dry season, high starch levels (*p* = 0.003) were recorded for clones CRG 0161/RED2, CRG 0228/105, and CRG 0328/216 (Table 2). Non‐structural carbohydrate levels increased in the leaves of 8 clones and decreased in 6 clones during the dry season compared to the wet season. This showed significant interaction between season and variety (*p* ≤ 0.001) and varietal significance (*p* = 0.003) changes (Tables 1 and 2). Non‐structural carbohydrate levels ranged from 36.00 mg g^−1^ (CRG 0412/113) to 74.90 mg g^−1^ (CRG 0128/201) in the wet season and at the dry season ranged from 33.70 mg g^−1^ in CRG 0412/113 to 86.73 mg g^−1^ in CRG 0161/RED2 (Table 2). The soluble sugars starch ratio levels detected in the leaves of the clones changed significantly in considering interaction between season and variety (*p* = 0.032) and seasonal effect (*p* ≤ 0.001). High values were recorded during the dry season compared to the wet season and ranged from 1.04 to 2.64 in CRG 0611/409 and CRG 0314/102 respectively (Table 2). Across varieties, the levels of non‐structural carbohydrates, soluble sugars, starch, and soluble sugar starch ratio were higher during the dry season compared to the wet season. For instance, the level of soluble sugars in the dry season was 36.32 mg g^−1^ compared to 25.29 mg g^−1^ recorded in the wet season across varieties (Tables SD4 and SD5).

**TABLE 2:** Soluble sugars, starch content, non‐structural carbohydrate and soluble sugars starch ratio of some clonal cocoa varieties in response to field stress conditions during vegetative phase.

Clonal cocoa varieties	Soluble sugar (mg g^−1^)		Starch (mg g^−1^)		NSC (mg g^−1^)		Soluble sugar starch ratio	
	WS	DS	WS	DS	WS	DS	WS	DS
CRG 0104/216	**20.66 ± 1.57bc**	**34.86 ± 6.46bc**	26.26 ± 4.15bc	24.50 ± 6.82abc	46.92 ± 2.81de	59.4 ± 13.25bc	0.91 ± 0.26a	1.56 ± 0.16 cd
CRG 0128/201	33.29 ± 1.40a	35.82 ± 5.80bc	**41.58 ± 3.89a**	**21.91 ± 4.99bcd**	**74.87 ± 4.77a**	**57.70 ± 10.70bc**	**0.82 ± 0.07a**	**1.78 ± 0.27bcd**
CRG 0132/105	27.59 ± 4.17ab	33.63 ± 2.75bc	**37.70 ± 1.19a**	**24.64 ± 3.73abc**	65.28 ± 4.36ab	58.27 ± 3.81bc	**0.73 ± 0.11a**	**1.47 ± 0.25 cd**
CRG 0145/205	**21.89 ± 3.96bc**	**33.23 ± 7.14bc**	26.05 ± 1.90bc	23.42 ± 4.92abc	47.94 ± 3.03 cd	56.65 ± 9.72bc	0.88 ± 0.23a	1.61 ± 0.49 cd
CRG 0161/RED2	**17.76 ± 2.72c**	**53.11 ± 6.15a**	**18.23 ± 1.20c**	**33.62 ± 2.15a**	**35.99 ± 1.60e**	**86.73 ± 8.13a**	**1.02 ± 0.22a**	**1.57 ± 0.11 cd**
CRG 0228/105	**17.38 ± 3.58c**	**40.79 ± 7.65ab**	26.05 ± 6.54bc	26.92 ± 7.36ab	**43.42 ± 7.73de**	**67.70 ± 15.00abc**	**0.78 ± 0.25a**	**1.77 ± 0.32bcd**
CRG 0314/102	**27.49 ± 4.22ab**	**36.06 ± 3.16b**	**41.16 ± 4.56a**	**13.87 ± 0.59 cd**	**68.65 ± 6.63ab**	**49.93 ± 2.56 cd**	**0.69 ± 0.13a**	**2.64 ± 0.33a**
CRG 0322/208	**24.66 ± 2.39ab**	**31.85 ± 2.09bc**	24.69 ± 4.26c	23.10 ± 3.34abcd	49.35 ± 3.60 cd	54.95 ± 5.26bcd	1.17 ± 0.37a	1.43 ± 0.13 cd
CRG 0322/214	**22.46 ± 4.39bc**	**34.24 ± 4.21bc**	27.14 ± 5.69bc	19.24 ± 2.91bcd	49.60 ± 1.62 cd	53.48 ± 2.73bcd	**1.09 ± 0.43a**	**1.94 ± 0.37abc**
CRG 0328/216	**22.15 ± 3.38bc**	**34.84 ± 5.97bc**	18.48 ± 1.98c	21.94 ± 1.75bcd	40.63 ± 5.30de	56.78 ± 7.07bc	1.18 ± 0.08a	1.58 ± 0.21 cd
CRG 0347/413	**29.19 ± 3.23ab**	**37.15 ± 2.25b**	**41.35 ± 4.81a**	**23.03 ± 1.89abcd**	70.54 ± 3.08ab	60.18 ± 4.14bc	**0.75 ± 0.14a**	**1.62 ± 0.04 cd**
CRG 0412/113	33.36 ± 3.57a	21.39 ± 5.55c	**25.92 ± 0.59bc**	**12.27 ± 2.59d**	**59.27 ± 3.31bc**	**33.66 ± 7.99d**	1.29 ± 0.16a	1.71 ± 0.15bcd
CRG 0611/409	27.61 ± 3.58ab	29.05 ± 3.18bc	35.82 ± 2.82ab	28.07 ± 1.74ab	63.43 ± 0.81ab	57.11 ± 4.23bc	0.81 ± 0.15a	1.04 ± 0.11d
T65/238	**28.51 ± 3.50ab**	**52.39 ± 4.29a**	**40.06 ± 1.75a**	**22.72 ± 2.98abcd**	68.57 ± 3.49ab	75.11 ± 2.14ab	**0.72 ± 0.09a**	**2.52 ± 0.58ab**

*Note:* Values are the means of 4 replicates with ±standard errors. Bolded values indicate significance (*p* = 0.005, student *t*‐test) between wet season (WS) and dry season (DS). Different letters indicate significant differences among the varieties within a season (Fisher pair wise comparison, *p* ≤ 0.05).

#### 3.1.4 Leaf Carbon‐Nitrogen Metabolism of Clonal Cocoa Varieties

The carbon and nitrogen as well as the carbon nitrogen ratio levels across varieties were comparable relative to seasons regarding maximum, median, minimum and means (Tables SD4 and SD5). Apart from nitrogen, high levels of carbon (*p* = 0.007) and C/N ratio (*p* = 0.001) were detected in the leaves of the clones during dry season compared to the wet season (Tables 1 and 3). The levels of carbon ranged from 31.78% in CRG 0314/102 to 35.51% in CRG 0412/113 during wet season (Table 3). The carbon again was low in CRG 0132/105 (33.20%) and high in CRG 0412/113 (35.39%) at the dry season. Nitrogen levels which were mainly high (*p* = 0.032) during the wet season ranged from 1.97% (CRG 0161/RED2) to 2.37% (CRG 0412/113) and in the dry season ranged from 1.91% to 2.24% in CRG 0611/409 and CRG 0347/413 respectively. The C/N was higher (17.06) in clone CRG 0161/RED2 and lower (15.34) in clone T65/238 at the wet season. In the dry season, the ratio increased in clones CRG 0611/409 (17.58), CRG 0128/201 (17.27), CRG 0161/RED2 (16.92) and CRG 0145/205 (16.90) and decreased in clones T65/238 (15.34), CRG 0132/105 (15.35), CRG 0328/216 (15.74) compared among the other clones (Table 3).

**TABLE 3:** Carbon, nitrogen content and carbon nitrogen ratio of some clonal cocoa varieties in response to field stress conditions during vegetative phase.

Clonal cocoa varieties	Carbon (DM%)		Nitrogen (DM%)		Carbon nitrogen ratio	
	WS	DS	WS	DS	WS	DS
CRG 0104/216	32.86 ± 1.21ab	34.47 ± 0.29a	2.31 ± 0.13a	2.18 ± 0.04ab	14.37 ± 0.85a	15.87 ± 0.61ab
CRG 0128/201	33.34 ± 0.67ab	34.47 ± 0.16a	**2.31 ± 0.14a**	**2.01 ± 0.08ab**	**14.58 ± 0.82a**	**17.27 ± 0.75ab**
CRG 0132/105	33.20 ± 1.97ab	33.20 ± 0.14a	2.27 ± 0.19ab	2.17 ± 0.10ab	15.07 ± 1.96a	15.35 ± 0.64b
CRG 0145/205	**33.81 ± 1.61ab**	**35.15 ± 0.40a**	2.13 ± 0.10ab	2.09 ± 0.13ab	15.97 ± 1.21a	16.90 ± 0.54ab
CRG 0161/RED2	**33.15 ± 0.76ab**	**34.71 ± 0.57a**	1.97 ± 0.15b	2.05 ± 0.08ab	17.06 ± 1.30a	16.92 ± 0.59ab
CRG 0228/105	**32.91 ± 1.09ab**	**33.78 ± 0.47a**	**2.23 ± 0.07ab**	**2.06 ± 0.11ab**	**14.83 ± 0.65a**	**16.47 ± 0.45ab**
CRG 0314/102	31.79 ± 1.00b	33.69 ± 0.20a	2.18 ± 0.07ab	2.08 ± 0.13ab	**14.66 ± 0.87a**	**16.39 ± 0.90ab**
CRG 0322/208	33.05 ± 0.75ab	33.93 ± 0.25a	2.10 ± 0.08ab	2.09 ± 0.08ab	15.76 ± 0.52a	16.35 ± 0.76ab
CRG 0322/214	**33.05 ± 1.35ab**	**34.81 ± 0.31a**	2.21 ± 0.07ab	2.10 ± 0.08ab	**15.12 ± 1.07a**	**16.71 ± 1.00ab**
CRG 0328/216	33.00 ± 0.98ab	33.78 ± 0.40a	2.21 ± 0.10ab	2.15 ± 0.06ab	15.03 ± 0.88a	15.74 ± 0.77ab
CRG 0347/413	33.54 ± 1.09ab	35.15 ± 0.29a	2.23 ± 0.06ab	2.24 ± 0.14a	15.04 ± 0.43a	15.87 ± 0.77ab
CRG 0412/113	35.52 ± 0.52a	35.39 ± 0.24a	**2.37 ± 0.09a**	**2.21 ± 0.08a**	15.06 ± 0.52a	16.12 ± 0.81ab
CRG 0611/409	33.44 ± 0.41ab	33.30 ± 0.25a	2.08 ± 0.04ab	1.91 ± 0.12b	**16.10 ± 0.31a**	**17.58 ± 0.80a**
T65/238	**32.37 ± 0.90b**	**34.27 ± 0.26a**	**2.12 ± 0.06ab**	**2.23 ± 0.04a**	15.34 ± 0.79a	15.34 ± 0.11b

*Note:* Values are the means of 4 replicates with ±standard errors. Bolded values indicate significance (*p* = 0.005, student *t*‐test) between wet season (WS) and dry season (DS). Different letters indicate significant differences among the varieties within a season (Fisher pair wise comparison, *p* ≤ 0.05).

### 3.2 Soft Selection Traits

#### 3.2.1 Plant Height and Stem Diameter Growth Rate of Clonal Cocoa Varieties

**FIGURE 4:**
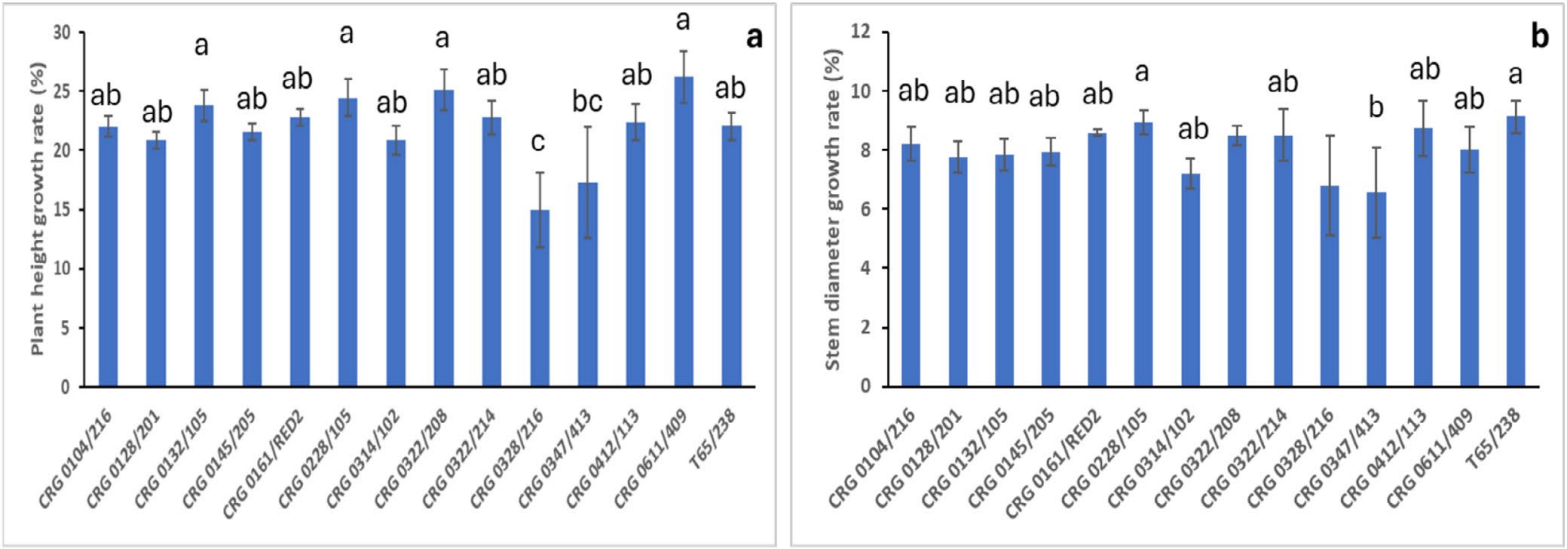
Growth rate of plant height (a) and stem diameter (b) of some clonal cocoa varieties in response to field stress conditions recorded at 35 and 44 months after planting (MAP). Values are the means of 4 replicates with standard error bars. The means were separated using Fisher pairwise comparison. Different letters indicate significant differences among the varieties.

The growth rate determined after 6 months interval (April to October) across the wet and dry seasons showed a significant difference (*p* = 0.001) in plant height among the varieties (Figure 4). The top three clones with greater plant height growth rates are CRG 0611/409 (29.21%), CRG 0322/208 (25.11%), and CRG 0228/105 (24.46%; Figure 4a). The clones' stem diameter growth rate ranged from 7.21% in CRG 0314/102 to 9.13% in T65/238 (Figure 4b). The maximum plant height growth rate observed across the varieties and seasons was 31.88%, median of 22.16%, minimum of 3.27%, with an average of 21.94%. While an average of 8.05%, maximum of 11.29%, median of 8.22%, and minimum of 2.09% were recorded for the stem diameter of the clones (Table SD1).

#### 3.2.2 Pod Number of Clonal Cocoa Varieties

Pod numbers observed between 35 and 44 months after planting showed significant difference among the varieties (*p* ≤ 0.001) and in seasons (*p* = 0.009, Tables 1 and 4). Clonal cocoa varieties CRG 0314/102, CRG 0145/205, T65/238, CRG 0132/105 and CRG 0347/413 are the five cocoa clone varieties which had the highest pod numbers ranging between 39 and 56 pods at wet season (Table 4). Clones CRG 0128/201 and CRG 0611/409 had low pod numbers of 14 and 16 respectively at wet season (Table 4). In the dry season the number of pods counted on the plants reduced drastically compared to the wet season. These ranged between 9 and 41 in clones CRG 0611/409 and CRG 0314/102 respectively. The top three clones (CRG 0132/105, CRG 0314/102 and T65/238) recorded in the dry season were also observed among the top five clones documented in the wet season (Table 4). The number of pods across varieties was higher in the wet season compared to dry season. An average of 32.98 and 25.57 number of pods were recorded during wet and dry seasons respectively with a maximum number of 71 (wet season) and 59 (dry season) pods. Minimum pods for the wet season were 5 while 2 for dry season with a median of 28 and 24 pods respectively (Table SD4).

**TABLE 4:** Number of pods of some clonal cocoa varieties in response to field stress conditions recorded in 2022 and 2023 wet and dry growing seasons between 35 and 44 months after planting.

Variety	Number of Pod	
	Wet season	Dry season
CRG 0104/216	35.75 ± 8.27abcde	25.50 ± 6.98abcd
CRG 0128/201	13.75 ± 2.06e	9.50 ± 0.89d
CRG 0132/105	40.80 ± 10.15abc	36.00 ± 9.16ab
CRG 0145/205	43.80 ± 12.60ab	29.00 ± 9.33abc
CRG 0161/RED2	18.25 ± 7.09cde	14.75 ± 6.27 cd
CRG 0228/105	23.00 ± 2.80bcde	20.50 ± 2.96bcd
CRG 0314/102	56.00 ± 6.87a	40.75 ± 7.21a
CRG 0322/208	38.00 ± 5.96abcd	33.50 ± 2.66ab
CRG 0322/214	33.50 ± 8.75abcde	23.50 ± 4.22abcd
CRG 0328/216	37.50 ± 7.08abcd	29.50 ± 5.46abc
CRG 0347/413	39.00 ± 13.25abcd	29.00 ± 8.36abc
CRG 0412/113	23.50 ± 8.42bcde	23.50 ± 8.21abcd
CRG 0611/409	15.75 ± 2.53de	9.00 ± 2.16d
T65/238	43.25 ± 8.67ab	34.00 ± 8.89ab

*Note:* Values are the means of 4 replicates with ±standard error. The means were separated using Fisher LSD method. Different letters indicate significant differences among the varieties.

### 3.3 The Relationship of All Parameters of Clonal Cocoa Varieties

Photochemical efficiency measured as Fv/Fm correlated positively or negatively with all the parameters except NSC and nitrogen content (Table 5). Relative water content positively correlated with stem diameter, plant height, and negatively with soluble sugar, soluble sugar starch ratio, carbon, and carbon nitrogen ratio. Soluble sugar correlated with NSC and soluble sugar starch ratio positively; however, the direction of correlation with plant height and stem diameter was negative. This negative direction also involved NSC, soluble sugar starch ratio, and carbon. Stem diameter and plant height significantly and positively correlated with number of pods (Table 5).

**TABLE 5:** Correlation between parameters evaluated during wet and dry growing cycles, values with asterisks indicate significance (*p* = 0.005).

	Fv/Fm	RWC	Gs	SS	S	NSC	SS/S	C	N	C/N	SD	PH
RWC	0.23*											
SS	−0.46*	−0.13	−0.36*									
S	0.34*	0.13	0.32*	0.12								
NSC	−0.11	−0.01	−0.05	0.78*	0.72*							
SS/S	−0.57*	−0.18*	−0.52*	0.55*	−0.66*	−0.04						
C	−0.24*	−0.21*	−0.18	0.14	−0.17	−0.01	0.26*					
N	0.15	0.15	0.24*	0.03	0.05	0.05	−0.01	0.08				
C/N	−0.26*	−0.25*	−0.30*	0.04	−0.14	−0.06	0.15	0.46*	−0.84*			
SD	0.86*	0.23*	0.68*	−0.49*	0.36*	−0.12	−0.58*	−0.24*	0.19*	−0.29*		
PH	0.86*	0.23*	0.68*	−0.48*	0.39*	−0.09	−0.59*	−0.24*	0.17	−0.26*	0.97*	
NP	0.20*	0.18	0.09	−0.14	0.13	−0.02	−0.09	0.09	0.10	−0.04	0.23*	0.19*

Abbreviations: C/N‐carbon to nitrogen ratio; C‐carbon; N‐nitrogen; NP‐Number of pods; NSC‐Non‐structural carbohydrate; PH‐plant height; RWC‐relative water content; SD‐stem diameter; SS/S‐soluble sugars to starch ratio; SS‐soluble sugars; S‐starch.

## 4 Discussion

The physiology and growth of cocoa plants is dependent on the changes of environmental conditions such as water and temperature (Asitoakor et al. 2022). Hard and soft selection traits revealed carbon central metabolism activity of the clonal cocoa varieties in response to varied environmental conditions. Non‐structural carbohydrates, soluble sugars, and starch accumulated as impacted by the field stress conditions during the dry season. These enhanced the observed relative water contents, which might have protected the clonal cocoa cellular membranes (Aidoo et al. 2016). The soil water levels determined in this study were higher at 40 cm depth than 10 cm depth throughout the growing season. Soil moisture levels and ambient temperatures reduced and increased, respectively, during the dry season compared to the wet season. This alters the photochemical efficiency (Fv/Fm), growth, and metabolism activity of the clonal cocoa varieties, reducing pod numbers. Hard and soft selection traits evaluated in this study showed relationships with each other (Table 4).

The significantly high levels of relative water content (RWC) observed in all the clones except CRG 0128/201 and CRG 0412/113 may suggest that stomatal closure during the dry season was a mechanism to preserve leaf water status and avoid oxidative stress (Haghpanah et al. 2024). Additionally, this mechanism might have contributed to the maintenance of leaf turgor that prevented tissue damage, ensuring osmotic adjustment (Medina and Laliberte 2017). Cocoa clones subjected to water deficit also revealed a similar result in maintained high levels of RWC (Almeida and Valle 2007), a mechanism that moderates membrane structural integrity when plants are exposed to environmental stress. The significant decrease of Fv/Fm in all the clones at dry season may reflect photoinhibition by oxidative stress (Zambrano et al. 2021) and low magnesium (Table SD2). This is in line with the finding of Zambrano et al. (2021) when they evaluated cocoa clones in response to water deficit. They reported that a drop in PSII quantum efficiency affects all clones during the photosynthetic limited phase which can eventually lead to plant death. This can be mitigated by increased carbohydrate concentration in plant roots (Sharma et al. 2020; Bertolde et al. 2012).

The high non‐structural carbohydrate annotated in this study may have activated plant photoprotective mechanisms, water homeostasis and metabolism maintenance (Nawaz et al. 2025). Rather than irreversible damage (Bhattacharjee and Saha 2014) of the clonal cocoa plants as indicated by the growth rates and physiological information (Figures 3 and 4). High levels of soluble sugars, starch and non‐structural carbohydrate were annotated in the leaves of clones CRG 0161/RED2, CRG 0228/105 and T65/238 during dry season. This finding agrees with the work done by Galvez et al. (2011) and Aidoo et al. (2024) on leaves of aspen and seed‐derived cocoa varieties under water stress and field stress conditions respectively. The accumulation of carbohydrate in the clones CRG 0161/RED2, CRG 0228/105 and T65/238 might have accounted for the high growth rate, Fv/Fm and relative water content in their response to field stress conditions (Figures 3 and 4). The accumulation of soluble sugars, starch and NSC may have also been used to maintain osmotic pressure and hydraulic functions. These functions are reported to protect cellular proteins from dehydration, growth enhancement and improvement of field stress tolerance of plants (O'Brien et al. 2014). In comparing the magnitude of accumulation of soluble sugars to starch (SS/S), high levels of soluble sugars were observed in all the clonal cocoa varieties indicating that when cocoa clones are subjected to extreme field conditions the plants amass soluble sugars in their leaves (Table 2 and Table SD4).

Interestingly, the levels of carbon and nitrogen were higher and lower in the dry season than wet season respectively and this was significantly different (Table 2). This indicates that the varieties' young leaves and roots' uptake rate of carbon, nitrogen and their utilization efficiency in response to dry season conditions was high (Njoroge et al. 2023). The high levels of carbon observed during dry season agree with the findings of Stefaniak et al. (2024). The group estimated optimal allocation of stored carbon over a single drought stress period and concluded that increased carbon storage during drought may be explained as an active process that enhances plant performance during stress. The low leaf nitrogen levels detected in the clonal cocoa varieties agree with a report on Kentucky bluegrass (Saud et al. 2017) and rapeseed cultivar (Biswas et al. 2019) response to drought and temperature stress. This may suggest plants' nitrogen use efficiency and/or catabolism to amino acids (Aidoo et al. 2017) which may have assuaged the extreme field stress conditions during dry season. Carbon nitrogen ratio increased at dry season (Table 2). This indicates that in clonal cocoa varieties carbon nitrogen ratio is unbalanced in response to dry season field conditions. This may be because of sugar accumulation detected in the leaves of the plants during dry season which may have favored the accumulation of carbon (Figure 4a). For instance, soluble sugars, particularly glucose and fructose, build up together with other osmolytes in response to drought to protect plants in environmental stress conditions (Chen et al. 2015). The continuous change of physiology and metabolism mechanisms of the clones in response to field stress conditions enhanced pod numbers particularly in clones CRG 0314/102, CRG 0132/105, T65/238 and CRG 0322/208 which is an indication of tolerance.

High growth rate was observed in the change of plant height of clones CRG 0611/409, CRG 0322/208 and CRG 0228/105 and stem diameter of clones T65/238, CRG 0228/105 and CRG 0161/RED2. Clone CRG 0228/105 was the only variety that had a high growth rate in both plant height and stem diameter (Figure 3). This finding agrees with a previous study on seed‐derived cocoa plants in response to field stress conditions (Aidoo et al. 2024) and might have been supported by the high nutrition of the soil (Table SD2). However, the growth reduction observed in the rest of the clonal cocoa varieties might be due to a decline in cell multiplication, enlargement and high leaf senescence because of the stress experienced in the field during the dry season (Yakoub et al. 2016).

The correlation analysis revealed either positive or negative high dependency of carbohydrate on all the parameters evaluated in this study (Table 4). The high positive and negative correlations that were observed between soluble sugars, relative water content and starch levels confirm the regulation of stomata and starch content by soluble sugars and relative water content (Dewar et al. 2022). This may have influenced pod yields in this study. Fv/Fm was positively associated with starch, nitrogen metabolism and the number of pods of clonal cocoa varieties in response to field stress and unstressed conditions. These findings fall in line with Aidoo et al. (2024) when they evaluated seed‐derived cocoa plants under field stress conditions. This is an indication that Fv/Fm is an important physiological parameter in predicting growth, metabolism and yield of clonal cocoa varieties subjected to stress and unstressed field conditions. A similar result has been presented by Sommer et al. (2023) in estimating yield performance after exposure of three wheat cultivars to drought stress during the anthesis stage.

The study highlights the importance of accumulation of carbon central metabolism. Reflecting on stress tolerant clonal cocoa varieties characterized by regulation of coordinated non‐structural carbohydrate metabolism, balanced carbon nitrogen relationships, stable photosynthetic efficiency and effective water management. The integration of these traits enables some clonal cocoa varieties to maintain metabolic activity and growth under field stress conditions. These findings provide valuable insights for cocoa breeding and selection programme highlighting the importance of physiological traits such as non‐structural carbohydrate partitioning, carbon nitrogen ratio, photochemical efficiency stability and water use efficiency as potential indicators for identifying stress resilient cocoa and other tree crop varieties.

## 5 Conclusion

The genetic variations of clonal cocoa varieties CRG 0132/105, CRG 0145/205, CRG 0314/102, and T65/238 agreed to the hypothesis, showing tolerance in responding to changing field stress conditions. The cocoa clones accumulated high levels of soluble sugars, starch, and non‐structural carbohydrate, improved photosynthetic efficiency, conserved cellular water, and enhanced growth rates during the dry season. These physiological and central carbon metabolism alterations were employed by some clones to assuage the extreme field stress conditions during the dry growing season to enhance productivity.

## Funding

The authors have nothing to report.

## Ethics Statement

The authors have nothing to report.

## Conflicts of Interest

The authors declare no conflicts of interest.

## Supporting information


**Figure SD1:** Monthly soil moisture at 10 and 40 cm depth of the experimental plot recorded before and during study. Arrows indicate the time point of taken data and sampling (Wet season‐June and Dry season January).

**Figure SD2:** Monthly ambient temperature and rainfall of the experimental plot recorded before and during study. Arrows indicate the time point of taken data and sampling (Wet season‐June and Dry season January).

**Figure SD3:** Total annual rainfall (a) and ambient temperature (b) of the study area recorded before, during and after the study.

**Table SD1:** Physical characteristics of soils sampled from 0 to 20 cm depth of the plot before the establishment of the trial. Values are means of three replicates with standard error.

**Table SD2:** Chemical characteristics of soils sampled from 0 to 20 cm depth of the plot before the establishment of the trial. Values are means of three replicates with standard error.

**Table SD3:** Soil physical and chemical properties across soil depth and type of selection.

**Table SD4:** Environmental factors and traits (across clones) measured during wet season showing maximum, median, minimum, mean, standard deviation and type of selection. A. Temp‐ambient temperature, Fv/Fm‐photochemical efficiency, RWC‐relative water content, NSC‐non‐structural carbohydrate, SS/S‐soluble sugar starch ratio. C/N‐carbon nitrogen ratio.

**Table SD5:** Environmental factors and traits (across clones) measured during dry season showing maximum, median, minimum, mean, standard deviation and type of selection. A. Temp‐ambient temperature, Fv/Fm‐photochemical efficiency, RWC‐relative water content, NSC‐non‐structural carbohydrate, SS/S‐soluble sugar starch ratio. C/N‐carbon nitrogen ratio.

## Data Availability

The data supporting the funding of this study are available within the article and its Supporting Information.
