# Supplementary material for: Clonal Cocoa Varieties Growth and Leaf Non‐Structural Carbohydrate Response to Field Stress Conditions
Source: Plant Environ Interact. 2026 May 13;7(3):e70160. doi: 10.1002/pei3.70160 (PMC13172295; doi:10.1002/pei3.70160)
Supplement: Supplementary file 1 — Figure SD1: Monthly soil moisture at 10 and 40 cm depth of the experimental plot recorded before and during study. Arrows indicate the time point of taken data and sampling (Wet season‐June and Dry season January). [file PEI3-7-e70160-s003.docx]

**
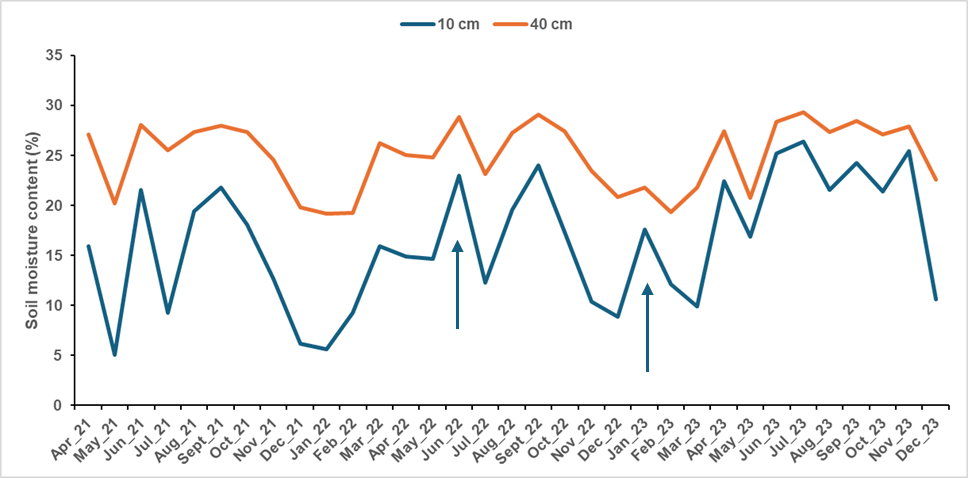
**

**FIGURE SD 1:** Monthly soil moisture at 10 and 40 cm depth of the experimental plot recorded before and during study. Arrows indicate the time point of taken data and sampling (Wet season-June and Dry season January).
