# Supplementary material for: Clonal Cocoa Varieties Growth and Leaf Non‐Structural Carbohydrate Response to Field Stress Conditions
Source: Plant Environ Interact. 2026 May 13;7(3):e70160. doi: 10.1002/pei3.70160 (PMC13172295; doi:10.1002/pei3.70160)
Supplement: Supplementary file 2 — Figure SD2: Monthly ambient temperature and rainfall of the experimental plot recorded before and during study. Arrows indicate the time point of taken data and sampling (Wet season‐June and Dry season January). [file PEI3-7-e70160-s007.docx]

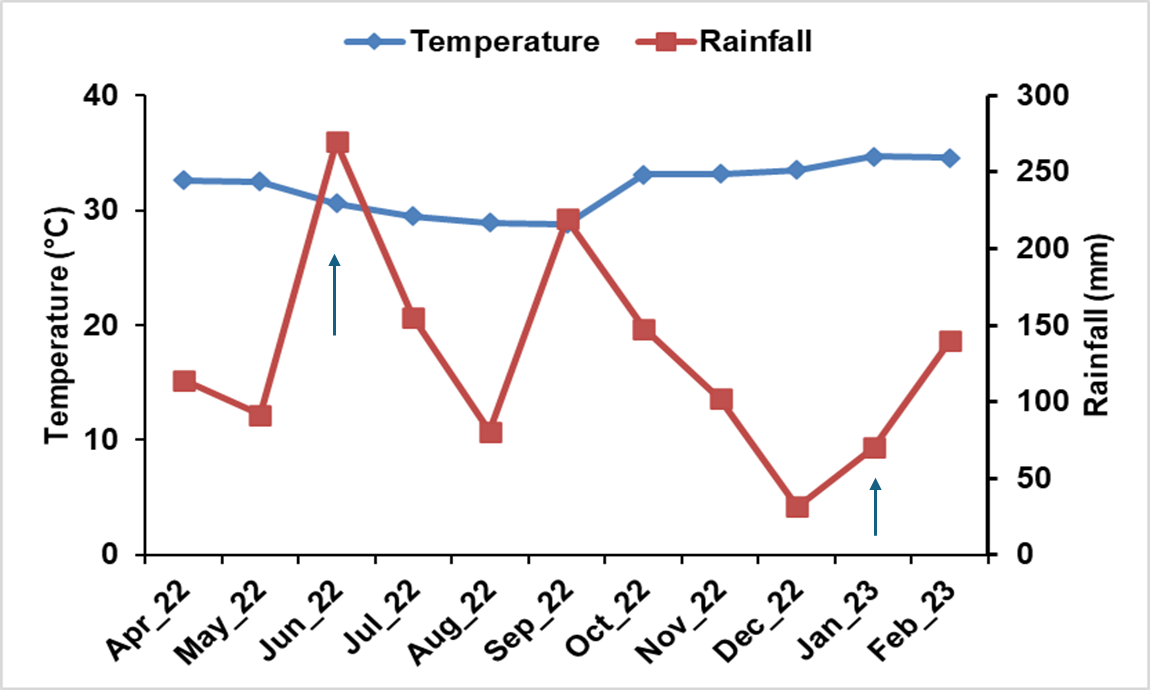


**FIGURE SD 2:** Monthly ambient temperature and rainfall of the experimental plot recorded before and during study. Arrows indicate the time point of taken data and sampling (Wet season-June and Dry season January).
