# Supplementary material for: Clonal Cocoa Varieties Growth and Leaf Non‐Structural Carbohydrate Response to Field Stress Conditions
Source: Plant Environ Interact. 2026 May 13;7(3):e70160. doi: 10.1002/pei3.70160 (PMC13172295; doi:10.1002/pei3.70160)
Supplement: Supplementary file 3 — Figure SD3: Total annual rainfall (a) and ambient temperature (b) of the study area recorded before, during and after the study. [file PEI3-7-e70160-s005.docx]

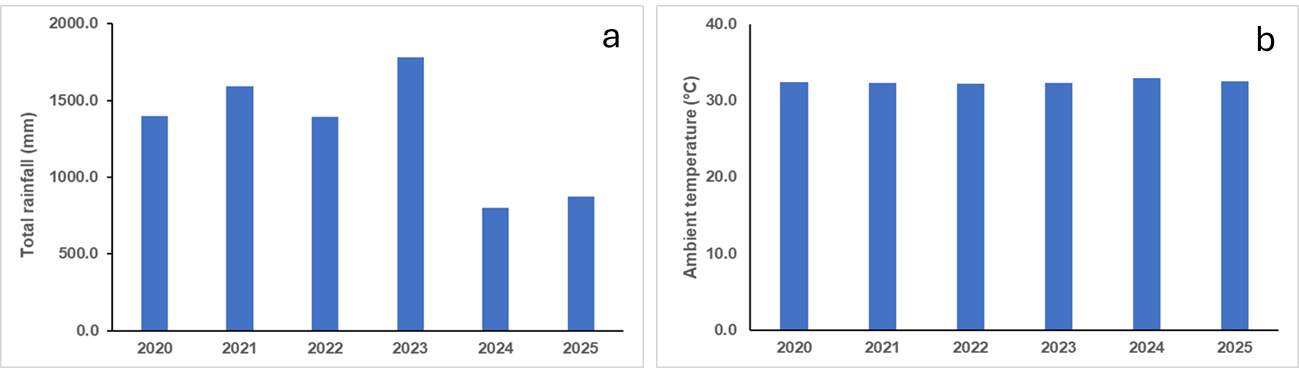


**FIGURE SD 3:** Total annual rainfall (a) and ambient temperature (b) of the study area recorded before, during and after the study.
