# Supplementary material for: Clonal Cocoa Varieties Growth and Leaf Non‐Structural Carbohydrate Response to Field Stress Conditions
Source: Plant Environ Interact. 2026 May 13;7(3):e70160. doi: 10.1002/pei3.70160 (PMC13172295; doi:10.1002/pei3.70160)
Supplement: Supplementary file 4 — Table SD1: Physical characteristics of soils sampled from 0 to 20 cm depth of the plot before the establishment of the trial. Values are means of three replicates with standard error. [file PEI3-7-e70160-s002.docx]

**TABLE SD 1:** Physical characteristics of soils sampled from 0 to 20 cm depth of the plot before the establishment of the trial. Values are means of three replicates with standard error.

| **Sand (%)** | **Clay (%)** | **Silt (%)** | **Textural Class** |
| --- | --- | --- | --- |
| 77.24 ± 0.94 | 13.76 ± 0.67 | 9.00 ± 0.67 | Sandy loam |
