# Supplementary material for: Clonal Cocoa Varieties Growth and Leaf Non‐Structural Carbohydrate Response to Field Stress Conditions
Source: Plant Environ Interact. 2026 May 13;7(3):e70160. doi: 10.1002/pei3.70160 (PMC13172295; doi:10.1002/pei3.70160)
Supplement: Supplementary file 5 — Table SD2: Chemical characteristics of soils sampled from 0 to 20 cm depth of the plot before the establishment of the trial. Values are means of three replicates with standard error. [file PEI3-7-e70160-s008.docx]

**TABLE SD 2:** Chemical characteristics of soils sampled from 0 to 20 cm depth of the plot before the establishment of the trial. Values are means of three replicates with standard error.

| **Factors** | **pH** | **Carbon**  **(%)** | **Total N**  **(%)** | **Avail P**  **(mg/kg)** | **Exch. K**  **(Cmol/kg)** | **Exch. Mg**  **(Cmol/ kg)** | **Exch. Ca**  **(Cmol/kg)** |
| --- | --- | --- | --- | --- | --- | --- | --- |
| Clone Plot | 5.84± 0.14 | 1.47±0.13 | 0.15±0.01 | 6.57±0.32 | 0.15±0.03 | 1.97±0.30 | 8.04±0.47 |
| Critical levels | 5.60 | 3.50 | 0.09 | 20.00 | 0.25 | 1.33 | 7.50 |
