# Supplementary material for: Clonal Cocoa Varieties Growth and Leaf Non‐Structural Carbohydrate Response to Field Stress Conditions
Source: Plant Environ Interact. 2026 May 13;7(3):e70160. doi: 10.1002/pei3.70160 (PMC13172295; doi:10.1002/pei3.70160)
Supplement: Supplementary file 6 — Table SD3: Soil physical and chemical properties across soil depth and type of selection. [file PEI3-7-e70160-s006.docx]

**Table SD 3:** Soil physical and chemical properties across soil depth and type of selection

| **Parameter** | **Maximum** | **Median** | **Minimum** | **Mean** | **Standard Deviation** | **Type of**  **Selection** |
| --- | --- | --- | --- | --- | --- | --- |
| Sand (%) | 79.24 | 77.24 | 75.24 | 77.24 | 1.63 | Hard |
| Clay (%) | 14.76 | 13.76 | 12.76 | 13.76 | 1.16 | Hard |
| Silt (%) | 10.00 | 9.00 | 8.00 | 9.00 | 1.16 | Hard |
| pH | 6.16 | 5.79 | 5.61 | 5.84 | 0.25 | Hard |
| Carbon (%) | 1.63 | 1.54 | 1.17 | 1.47 | 0.23 | Hard |
| Total N (%) | 0.16 | 0.15 | 0.12 | 0.15 | 0.02 | Hard |
| Avail. P (mg/kg) | 7.26 | 6.48 | 6.05 | 6.57 | 0.55 | Hard |
| Exch. K (cmol/kg) | 0.22 | 0.15 | 0.09 | 0.15 | 0.05 | Hard |
| Exch Mg (cmol/kg) | 2.45 | 2.08 | 1.27 | 1.97 | 0.52 | Hard |
| Exch. Ca (cmol/kg) | 8.70 | 8.30 | 6.86 | 8.04 | 0.81 | Hard |
