# Supplementary material for: Clonal Cocoa Varieties Growth and Leaf Non‐Structural Carbohydrate Response to Field Stress Conditions
Source: Plant Environ Interact. 2026 May 13;7(3):e70160. doi: 10.1002/pei3.70160 (PMC13172295; doi:10.1002/pei3.70160)
Supplement: Supplementary file 7 — Table SD4: Environmental factors and traits (across clones) measured during wet season showing maximum, median, minimum, mean, standard deviation and type of selection. A. Temp‐ambient temperature, Fv/Fm‐photochemical efficiency, RWC‐relative water content, NSC‐non‐structural carbohydrate, SS/S‐soluble sugar starch ratio. C/N‐carbon nitrogen ratio. [file PEI3-7-e70160-s001.docx]

**TABLE SD 4**: Environmental factors and traits (across clones) measured during wet season showing maximum, median, minimum, mean, standard deviation and type of selection. A. Temp-ambient temperature, Fv/Fm-photochemical efficiency, RWC-relative water content, NSC-non-structural carbohydrate, SS/S-soluble sugar starch ratio. C/N-carbon nitrogen ratio.

| **Parameter** | **Maximum** | **Median** | **Minimum** | **Mean** | **Standard Deviation** | **Type of**  **Selection** |
| --- | --- | --- | --- | --- | --- | --- |
| Soil Moisture (%) | 29.33 | 24.52 | 12.30 | 23.67 | 4.80 | Hard |
| Fv/Fm | 0.72 | 0.60 | 0.44 | 0.60 | 0.06 | Hard |
| RWC | 85.67 | 81.44 | 77.80 | 81.49 | 1.75 | Hard |
| Soluble Sugar | 43.56 | 25.28 | 10.43 | 25.29 | 7.73 | Hard |
| Starch | 53.65 | 30.40 | 13.47 | 30.75 | 10.50 | Hard |
| NSC | 87.48 | 55.19 | 27.03 | 56.03 | 14.25 | Hard |
| SS/S | 2.27 | 0.80 | 0.25 | 0.92 | 0.43 | Hard |
| Carbon | 38.22 | 33.35 | 28.86 | 33.22 | 2.08 | Hard |
| Nitrogen | 2.80 | 2.16 | 1.68 | 2.19 | 0.21 | Hard |
| C/N | 20.13 | 15.37 | 11.34 | 15.28 | 1.82 | Hard |
| Plant Height (cm) | 31.88 | 22.16 | 3.27 | 21.94 | 4.45 | Soft |
| Stem diameter (mm) | 11.39 | 8.22 | 2.09 | 8.05 | 1.61 | Soft |
| Number of Pod | 71.00 | 28.00 | 5.00 | 32.98 | 18.45 | Soft |
