# Supplementary material for: Clonal Cocoa Varieties Growth and Leaf Non‐Structural Carbohydrate Response to Field Stress Conditions
Source: Plant Environ Interact. 2026 May 13;7(3):e70160. doi: 10.1002/pei3.70160 (PMC13172295; doi:10.1002/pei3.70160)
Supplement: Supplementary file 8 — Table SD5: Environmental factors and traits (across clones) measured during dry season showing maximum, median, minimum, mean, standard deviation and type of selection. A. Temp‐ambient temperature, Fv/Fm‐photochemical efficiency, RWC‐relative water content, NSC‐non‐structural carbohydrate, SS/S‐soluble sugar starch ratio. C/N‐carbon nitrogen ratio. [file PEI3-7-e70160-s004.docx]

**TABLE SD 5:** Environmental factors and traits (across clones) measured during dry season showing maximum, median, minimum, mean, standard deviation and type of selection. A. Temp-ambient temperature, Fv/Fm-photochemical efficiency, RWC-relative water content, NSC-non-structural carbohydrate, SS/S-soluble sugar starch ratio. C/N-carbon nitrogen ratio.

| **Parameter** | **Maximum** | **Median** | **Minimum** | **Mean** | **Standard Deviation** | **Type of**  **Selection** |
| --- | --- | --- | --- | --- | --- | --- |
| Soil Moisture (%) | 27.84 | 19.27 | 5.63 | 18.23 | 6.48 | Hard |
| Fv/Fm | 0.55 | 0.37 | 0.27 | 0.37 | 0.06 | Hard |
| RWC | 93.95 | 80.83 | 20.62 | 75.21 | 18.28 | Hard |
| Soluble Sugar | 63.69 | 35.17 | 11.21 | 36.32 | 11.97 | Hard |
| Starch | 42.00 | 23.08 | 7.40 | 22.80 | 8.59 | Hard |
| NSC | 98.46 | 55.91 | 18.61 | 59.12 | 18.19 | Hard |
| SS/S | 4.13 | 1.57 | 0.77 | 1.73 | 0.65 | Hard |
| Carbon | 37.83 | 34.22 | 29.84 | 34.29 | 1.79 | Hard |
| Nitrogen | 2.45 | 2.16 | 1.67 | 2.11 | 0.19 | Hard |
| C/N | 19.69 | 16.06 | 13.75 | 16.35 | 1.41 | Hard |
| Number of Pod | 59.00 | 24.00 | 2.00 | 25.57 | 14.60 | Soft |
